# The Future of Flu: A Review of the Human Challenge Model and Systems Biology for Advancement of Influenza Vaccinology

**DOI:** 10.3389/fcimb.2019.00107

**Published:** 2019-04-17

**Authors:** Amy Caryn Sherman, Aneesh Mehta, Neal W. Dickert, Evan J. Anderson, Nadine Rouphael

**Affiliations:** Department of Medicine, Division of Infectious Diseases, Emory University, Atlanta, GA, United States

**Keywords:** human challenge model, influenza vaccination, systems biology, universal influenza vaccination, bioethics, transcriptomics, metabolomics

## Abstract

**Objectives:** Novel approaches to advance the field of vaccinology must be investigated, and are particularly of importance for influenza in order to produce a more effective vaccine. A systematic review of human challenge studies for influenza was performed, with the goal of assessing safety and ethics and determining how these studies have led to therapeutic and vaccine development. A systematic review of systems biology approaches for the study of influenza was also performed, with a focus on how this technology has been utilized for influenza vaccine development.

**Methods:** The PubMed database was searched for influenza human challenge studies, and for systems biology studies that have addressed both influenza infection and immunological effects of vaccination.

**Results:** Influenza human challenge studies have led to important advancements in therapeutics and influenza immunization, and can be performed safely and ethically if certain criteria are met. Many studies have investigated the use of systems biology for evaluating immune response to influenza vaccine, and several promising molecular signatures may help advance our understanding of pathogenesis and be used as targets for influenza interventions. Combining these methodologies has the potential to lead to significant advances in the field of influenza vaccinology and therapeutics.

**Conclusions:** Human challenge studies and systems biology approaches are important tools that should be used in concert to advance our understanding of influenza infection and provide targets for novel therapeutics and immunizations.

## Introduction

Although influenza virus was recognized as an important pathogen over a century ago, influenza continues to cause a significant burden of disease. In the United States alone, it's estimated that in the 2017–2018 season there were 959,000 hospitalizations related to influenza illness, and 79,400 deaths (CDC, 2018). Worldwide, WHO estimates that annual influenza epidemics cause 3–5 million cases of severe disease, with 290,000–650,000 of these severe cases resulting in death (Influenza (Seasonal), 2018). Although annual influenza immunizations are recommended and antivirals are available, both have several limitations. The efficacy of the seasonal influenza vaccine is compromised by several factors: antigenic changes over time (requiring a strain specific match each year), slow manufacturing processing, vaccine strain egg-adapted changes, short duration of protection, lack of cross-reactivity, and poor immunogenicity in certain populations (e.g., the elderly) (Goodwin et al., 2006; Soema et al., 2015; Raymond et al., 2016). Antiviral agents such as neuraminidase inhibitors are most effective if administered early in the disease course, and even then have only a modest impact upon the duration of clinical symptoms (McNicholl and McNicholl, 2001). Furthermore, data are inconclusive regarding the ability of neuraminidase inhibitors to reduce the risk of complications, such as hospitalizations or progression to pneumonia (Doll et al., 2017).

Novel platforms to understand influenza immunology are essential in order to address the burden of influenza disease and develop a more effective influenza vaccine that does not rely on annual updates. Combining old modalities—human challenge studies—with new technology—systems biology—has the potential to lead to exciting discoveries that can achieve this goal. There are many reasons why human challenge studies are essential for scientific progress, especially for the influenza field. Human challenge models have several benefits over traditional models such as animal and epidemiological models. Although mice and ferrets have been used in influenza challenges, animal models do not directly translate to humans in regards to their baseline immunity and subsequent immunological responses. Epidemiological or field studies have also been applied to study influenza vaccine efficacy, such as Petrie et al who followed a cohort of over 1,000 individuals from 2014 to 2015 to assess vaccine effectiveness (Petrie et al., 2017). However, these studies require a large sample size and often require numerous sampling points and several years to acquire sufficient data, with many conflicting and cofounding variables. In contrast, the human challenge model is efficient (relatively few participants are required to power a study), immunological responses of humans can be studied directly, and the exact timing of infection is known so that specific time points and measurements are precisely determined.

Human challenge studies for influenza are particularly attractive, with current national emphasis on development of a universal influenza vaccine. As outlined by NIAID's strategic plan, a universal flu vaccine would be at least 75% effective, maintain protection for at least 1 year, protect against group I (e.g., H1, H5) and II (e.g., H3, H7) influenza A virus strains, and be effective for all age groups (Erbelding et al., 2018). The strategic plan also elucidated how a human challenge model could offer unique benefits to better understand the concept of imprinting, determine correlates of protection against influenza, and evaluate different universal influenza vaccine candidates.

In this review, we will examine influenza human challenge studies that have been conducted and their safety, as well as review the ethical considerations in designing a challenge model. We will also review how the use of systems biology techniques in the context of human influenza challenge studies has great potential to advance our current understanding of the host response to acute influenza infection, and ultimately aid in the development of a universal influenza vaccine. The PubMed database was used to search for relevant clinical trials related to these subjects. We propose that successful integration of the right model (the human challenge study) in combination with systems biology approaches will help to better understand the immunological mechanisms of influenza infection and effects of vaccination, which will ultimately aid in the development of an improved vaccine (and perhaps even a universal influenza vaccine), and novel influenza therapeutics.

### History and Safety of Influenza Challenge Models

In medical research, there is a long and complex history of human challenge studies, in which healthy participants are intentionally infected in order to study the natural history of a disease or to test experimental therapeutic and preventative measures. Perhaps the most famous challenge study in infectious disease was Edward Jenner's use of cowpox in 1796. Although he is not the first to use intentional infection to protect against disease—historical records show that similar practices were likely occurring in Africa, India and China (Gross and Sepkowitz, 1998) long before the eighteenth century—he was responsible for publishing and popularizing the idea. Jenner inoculated the 8-year-old boy James Phipps with cowpox derived from a lesion on the hand of a dairymaid, Sarah Nelms (Riedel, 2005). This successful challenge lead to the creation of the first vaccine, with widespread use in Europe by the year 1800, and eventual eradication of smallpox.

The first well described influenza challenge study was published by Smorodintseff et al. in 1937. The authors infected 72 volunteers via inhalation of a human influenza virus, that had been maintained through the passage of ferrets and mice (Smorodintseff et al., 1937). They discovered that only a small percent (~20%) developed disease that was mild in intensity. The model was deemed safe and was utilized for several decades thereafter to understand immune responses to influenza and test preventative and therapeutic measures.

However, in the early 2000s, influenza human challenge studies came to a halt. This was a direct result of an adverse outcome associated with a human challenge study that was investigating the use of peramivir as a prophylactic agent (Ison et al., 2005). The subject was a 21-year-old, previously healthy individual with no prior cardiac history. Following the infection with mild influenza B infection and receipt of peramivir, he had asymptomatic ECG changes (described as new T wave inversions of leads II, aVF, and v4-6) on day 4 of the study. Repeat ECG at day 15 had returned to baseline, and he had no cardiac symptoms or enzyme elevation of CPK. He then traveled to Indonesia for 2 weeks, and became ill with an URI. When he returned to the US, he had an echocardiography performed 51 days after challenge since he had had initial ECG changes earlier. He had new reduced ejection fraction, with left ventricular enlargement and remained asymptomatic. Extensive work-up was unrevealing for infectious etiologies. Over the next 5 months, repeat echocardiograms showed gradual improvement and return to normal ejection fraction.

Despite the subject's return to baseline health, and despite the lack of direct causality linking myocarditis to the influenza challenge stock, no further influenza challenge studies were conducted in the US for nearly a decade later. Internationally, as well, only a few challenge studies for influenza were conducted in this time period. After the H1N1 pandemic, Memoli et al. at the NIH re-visited the human challenge model with their validation of a A/H1N1 challenge strain (Memoli et al., 2015). His group challenged 46 healthy participants with a virus that was rescued using reverse genetics (A/CA/04/2009) , with the goal to determine the dose needed to produce mild to moderate influenza infection in at least 60% of the participants who had baseline HAI titers of ≤1:40. The experimental influenza virus was delivered intranasally, and the participants were kept in isolation for at least 9 days following the challenge. The participants' symptoms were monitored and their immune response (serum cytokines, HAI and neuraminidase inhibition titers) documented at specific pre- and post-challenge time points. Discharge from isolation occurred only after the participant had two negative nasal washes on consecutive days. All of the participants demonstrated clinical symptoms of infection (as intended), and 70% of participants had both viral shedding and symptoms. At the dose of 10^7^ TCID_50_, 85% of the participants who received that dose had a ≥4-fold rise in HAI titer by week 8. No significant adverse effects or complications of the influenza infection occurred. Carrat et al. also performed a large and thorough review of 56 different influenza challenge studies, and confirmed that infection from a challenge stock induced only mild disease, with one third of participants having a fever and one fifth of participants developing lower respiratory symptoms (Carrat et al., 2008). This extensive review, in addition to further studies by Memoli's team (Memoli et al., 2016; Hunsberger and Memoli, 2017; Han et al., 2018), demonstrated that influenza challenges can be implemented safely and used in influenza research.

### Ethical Considerations for Human Challenge Studies

Human challenge studies raise specific ethical concerns because they place research subjects at risk with no potential for direct benefit. The main considerations that must be weighed include the level of risk to the participants, the overall social value of the study, the absence of good alternative study designs, and informed consent.

In human subject research, the risk-to-benefit ratio is usually favorable to the subject, before the risk-to-benefit ratio is weighed for the broader society (Pollard et al., 2012). For example, an individual with a rare and life-threatening disease may choose to participate in a trial that tests a novel therapy, because they personally may benefit from a cure for an otherwise untreatable condition. However, for the human challenge model, the participant is not directly gaining anything for their health, and the risk-to-benefit ratio leans toward the side of risk. Therefore, an acceptable challenge model must have risks that are reasonable to the participants, because the challenge model can be justified only by the benefits to society and not to the individuals. To mitigate risk to the participants, certain criteria should be evaluated. The study hypothesis must only be answerable by challenging human subjects; if the question and endpoint of the study could be determined by animal models or *in vitro* techniques, the human challenge model should not be used. Infected subjects should also have therapy available (in the case of influenza, antivirals such as oseltamivir or peramivir) in the event that they develop severe influenza infection during the experimental challenge. Participants should receive compensation for their time and effort; however, the more controversial topic is how much participants should receive. Is there a certain amount that is too much, and thus coercive? And likewise, is there an amount that is too little based on the risk inherent in the challenge study? Several authors, such as Miller and Grady, have offered a framework to evaluate the ethical considerations of an infection-inducing challenge study (Miller and Grady, 2001). To maintain ethical integrity, other specific considerations must be addressed in designing an influenza human challenge study, as outlined in Table 1.

**Table 1:** Ethical considerations in the design of an influenza challenge model.

Selection of the appropriate strain and dose of influenza challenge stock to achieve mild to moderate influenza illness.
Strict inclusion and exclusion criteria to ensure healthy volunteers with minimal comorbidities.
Full review of the proposed study by a third-party ethics committee.
Selection of appropriate clinical and microbiological endpoints to minimize risk to participants.
Transparent informed consent and fair compensation for participants.
Facilities and trained staff that can ensure close and careful monitoring of infected participants.
Proof of decreased infectivity (e.g., undetectable virus by molecular testing) upon discharge to eliminate possibility of transmission to general public.
Adequate clinical follow-up and evaluation for adverse events or sequelae of influenza infection.

^*^Loosely adapted from Darton et al. (2015) and Miller and Grady (2001).

If these criteria and considerations are carried out in a thoughtful manner, influenza challenge studies can be implemented safely and ethically.

A limitation of the human challenge model is the requirement to have healthy volunteers with no significant comorbidities. As stated above, healthy volunteers must be recruited in order to be ethically sound, in order to reduce the overall risk and complications to the individual. However, this limits our understanding of influenza pathogenesis and vaccine performance in high-risk populations who have the greatest likelihood of severe influenza complications, such as the very young and elderly, immunocompromised, and those with comorbidities. Another inherent limitation of this model is the goal to produce only mild to moderate influenza disease, not severe disease. Again, the efficacy of new therapeutics would be most important in the population suffering severe disease, which cannot be tested in this model for ethical reasons.

### Advancing Influenza Therapeutics With the Influenza Human Challenge Model

The influenza challenge model has proven useful in advancing the development of several current antivirals to the market, while also useful in terminating the clinical development of others. Two large randomized controlled trials were conducted in 1997 by Hayden et al. which investigated the use of oseltamivir, an oral neuraminidase inhibitor, for both prophylaxis and treatment (Hayden et al., 1999). Healthy participants (*N* = 117) were inoculated with influenza A/Texas/36/91 (H1N1), and given oseltamivir (at two different doses) or placebo. The results showed that prophylaxis and early treatment with oseltamivir significantly reduced symptoms and had anti-viral effects. This trial is the basis for the standard of care practiced today—oseltamvir continues to be recommended for influenza prophylaxis and treatment by clinical treatment guidelines (Uyeki et al., 2019). Peramivir, another neuraminidase inhibitor agent, was also tested using a similar design. Four randomized, double-blind, placebo-controlled trials were conducted, with 288 healthy volunteers inoculated with either A/Texas/36/91 (H1N1) or B/Yamagata/16/88 (Barroso et al., 2005). At the time of the study, oseltamivir was the only oral neuraminidase inhibitor available. Therefore, the authors sought to determine the tolerability and antiviral efficacy of oral peramivir for treatment and prophylaxis of influenza A and B, using the experimental influenza virus. The results showed that an oral dose of 200 mg twice daily or 400 mg once a day was effective for influenza A, and that prophylaxis with peramivir did not significantly reduce viral shedding. The authors also found relatively low blood peramivir concentrations, and recommended pursuing further study with parental dosing. In both the oseltamivir and peramivir studies, the challenge model was essential for understanding the exact timing of infection in order to test the efficacy of the new agents and identify the appropriate dosing schedule.

Testing of therapies that did not work were equally important in advancing the field. For example, a topical interferon inducer was tested in subjects experimentally infected with an influenza A H3N2 strain (Douglas et al., 1975). They found no difference in frequency or severity of illness, or in quantitative viral shedding between the placebo group and the group who received the interferon inducer. Another study investigated a new antiviral (agent ICI 130,685) that was similar to amantadine for both prophylactic and therapeutic use (Al-Nakib et al., 1986). The authors found that that the higher dosage of 200 mg/day of the new agent did have protective efficacy as compared to placebo when used for prophylaxis; however, the number of side effects in this group were double the side effects reported for the placebo group. For the therapeutic group, the new agent did show a reduced mean daily clinical score and decreased virus in the nasal washings as compared to the placebo arm. Yet, these reductions were not statistically significant for viral concentration until day 3, and for symptom score until day 4. Therefore, this product did not forward toward licensure since the risks (increased side effects) were greater than the benefits (only minor reductions in viral shedding and late symptom improvement).

### Advancing Vaccine Development With the Influenza Challenge Model

Human challenge studies have also been critical for the development of influenza vaccines. The first influenza vaccines were developed and distributed in the US in 1938, and provided to soldiers in World War II (Hannoun, 2013). Thomas Francis, who was a leader in the field of influenza and the author of “Doctrine of Original Antigenic Sin,” published a small trial in 1940 in which he inoculated active influenza viral cultures into the nares of 11 human subjects (Francis, 1940). He reported that none of the subjects experienced significant signs or symptoms of infection, and one individual showed a rise of influenza antibodies. He proposed that this technique may have potential for vaccine development. Another study in 1942–1943 challenged individuals with active influenza virus who had received an allantoic fluid vaccine at least 4 months before (Henle et al., 1943). Although it was a small study, the investigators were able to determine the efficacy of the vaccine. Of their controls, ten of twenty-eight individuals developed clinical influenza after inhalation of the isolated and active virus. In contrast, only one of forty-four individuals became ill with influenza after receiving the vaccination. Other early investigations had similar goals, and tested vaccine efficacy for both influenza A and B (Francis and Magill, 1937; Francis et al., 1945; Henle et al., 1946). In 1971, Couch et al. investigated the use of a recombinant influenza A vaccine (X-31 influenza A_2_, Hong Kong variant) in comparison to the standard vaccine (Couch et al., 1971). Two groups of healthy volunteers were given either the recombinant or the standard vaccine, and then challenged with the live virus strain (Hong Kong variant) used in the vaccine a month later. They measured neuraminidase-inhibiting antibody in the serum, neutralizing antibody in nasal secretions, influenza virus in the nasal secretions, and degree of clinical illness. The results demonstrated that the two vaccines were equally effective. Treanor et al. later challenged healthy adults with wild-type influenza strains to compare the efficacy of a trivalent, live, cold-adapted influenza vaccine (CAIV-T) against the trivalent inactivated influenza vaccine (TIV) (Treanor et al., 1999). After challenging the immunized individuals, they measured influenza illness (defined as respiratory symptoms with wild-type influenza virus isolated from nasal passages or >4-fold increase of HAI antibody from baseline). The efficacy of the immunizations was calculated as 85% for CAIV-T and 71% for TIV, although the difference was not statistically significant.

## Immunology and Applying Systems Biology to Influenza Vaccinology

### Pathogenesis and Immunology of Influenza Infection

Several studies have used experimental influenza inoculation of humans to investigate the pathogenesis and immune mechanisms associated with infection. Many sought to better understand viral infectivity by route of transmission (e.g., aerosolization), or determine if influenza could be transmitted across different host species (Kasel et al., 1965). For example, one study sought to determine if equine influenza virus could produce clinical symptoms if given to a human (Alford et al., 1966).

Others investigated various human immune responses following experimental infection. The mechanism of systemic and local antibody responses to infection was examined in a study conducted by Murphy et al. Their volunteers were children, aged 1.5 to 4.5 years old, and were inoculated intranasally with either A/Alaska/7/77 (H3N2) or A/Hong Kong/123/77 (H1N1) viruses (developed as candidate vaccine viruses) (Murphy et al., 1982). They found that there was a correlation between the IgA HA antibodies recovered from the nasal passageways and the serum IgG response. Thus, they proposed that intranasal inoculation stimulates both systemic and local immune responses and could be used for immunization purposes. Brown et al. sought to further explore the distinction between serum and secretory IgA antibodies in response to infection. By challenging 13 human volunteers with intranasal A/Peking/2/79(H3N2) wild-type virus, they measured serum and nasal IgA antibodies and their subclasses pre- and post- inoculation with the influenza virus (Brown et al., 1985). They found that IgA1 accounted for most of the increase in IgA anti-HA levels after infection, and determined that the origin of serum IgA antibodies to HA were from the mucosa.

Other investigations examined cell-mediated immune responses, challenging healthy volunteers with different strains of influenza A and measuring serum antibody, viral shedding, and other peripheral blood parameters (such as white blood cell counts), as well as local and systemic cytokine responses. In 1977, Dolin et al administered influenza A virus to 19 volunteers in order to assess cell-mediated immune responses up to 4 weeks after the challenge (Dolin et al., 1977). Eight of the nineteen volunteers had clinical symptoms and 4-fold increases of serum antibody, and lymphopenia was described in this cohort. The authors found that depression of lymphocytes and decreased functionality of the lymphocytes were present even at 4 weeks post-challenge. Another study by Hayden et al challenged volunteers with a H1N1 influenza strain and examined the relationship between clinical symptoms and cytokine responses (Hayden et al., 1998). They collected nasal lavage fluids, plasma and serum levels from the participants and analyzed various cytokines (IL-1 β, IL-2, IL-6, IL-8, IFN-α, TGF-β, and TNF-α) over time. The authors described an association between nasal fluid IFN-α, IL-6, and TNF-α levels and fever on day 2 post-challenge, and IFN-α and IL-6 levels with lower respiratory symptoms on days 5 and 6. They found that IL-6 levels were associated with total, systemic, and upper respiratory symptom scores on days 2 and 4. They concluded that IL-6 is likely the main factor involved in causing fever in influenza (not TNF-α, which is usually described as contributing to fever symptoms in other infections) based on the larger magnitude of IL-6 response early in the course of infection, and IFN-α is responsible for early systemic and local symptoms experienced in influenza infection. The authors hoped that their description of the cytokine response to influenza could be utilized to either (a) develop therapeutic agents that would target specific cytokine responses, or (b) use cytokine levels to more accurately measure the impact of an antiviral intervention. An interesting study by Gentile et al. utilized the influenza human challenge model to control for inflammatory responses seen in patients with allergic rhinitis, which they hypothesized would cause more severe disease (Gentile et al., 2001). Gentile et al enrolled 27 participants who had a history of allergic or nonallergic rhinitis, and then inoculated the participants with an influenza A H1N1 strain and measured anti-IgE-induced leukocyte histamine release, plasma histamine levels, and serum IgG, IgA, IgM, and IgE. The results showed no significant enhancement of systemic immune or inflammatory responses after inoculation in the allergic rhinitis group. The authors speculated that perhaps no difference was observed because the experimental infection was so mild in nature.

### Systems Biology and Influenza Immunization

The field of systems biology applied to vaccines uses mathematical modeling, networking, and other measurements to describe and predict the human immune response to vaccines. In addition, systems biology approaches have the potential to better explain the host-viral interaction and elucidate specific immunological pathways and mechanisms in regards to both influenza natural infection and influenza vaccination. Using systems biology methodologies may be especially useful in the context of influenza human challenge studies.

Nakaya et al. have done extensive work describing specific molecular signatures in individuals who have received seasonal influenza vaccines (Nakaya et al., 2011). They compared immune responses in individuals who had received either the trivalent inactivated vaccine (TIV) or live attenuated influenza vaccine (LAIV). They identified molecular signatures that correlated with B cell responses at day 7 and 28 after immunization, and demonstrated how these could be used to predict an individual's later immune response to the vaccine. Several specific genes were identified that corresponded with HAI response, many of which were regulated by XBP-1 (transcription factor). XBP-1 has been shown to be necessary for the differentiation of plasma cells and the unfolded protein response (Iwakoshi et al., 2003). Another important feature of an effective vaccine is its ability to produce and maintain a long-term durable antibody response to provide long-lasting protection. In a separate study, Nakaya et al. investigated antibody responses after influenza vaccination at day 28 vs. 180, with emphasis on identifying molecular pathways associated with either a persistent or waning antibody response with time (Nakaya et al., 2015). One potentially important pathway associated with persistent antibody response involves P-selectin (SELP), which affects mobility of leukocytes to the vaccine administration site from the peripheral blood stream. The authors were also able to identify transcriptional responses to vaccination, with particular interest in micro RNA expression of miR-424. MiR-424 is a regulator of the interferon response that occurs post vaccination. Another contribution was the finding of specific signatures associated with different age groups. The authors demonstrated that in the elderly population, modules associated with antiviral and IFN-related genes were impaired in the early innate response as compared to the younger population. In the elderly population, there was an enhanced NK-cell related expression and higher proportions of monocytes at baseline and post vaccination. It is hypothesized that perhaps these persistent inflammatory responses seen in the elderly may actually be having a negative effect by inhibiting appropriate vaccine immune responses, and perhaps explains the underlying mechanism of immunosenescence.

Several other studies have examined patterns of gene expression in response to influenza vaccination. The results of key experiments are summarized in Table 2.

**Table 2:** Using systems biology to study immune responses to influenza vaccination.

**Study title (first author)**	**Year**	**Key molecular correlate(s) identified**	**Contribution**
Systems analysis of immunity to influenza vaccination across multiple years and in diverse populations reveals shared molecular signatures (Nakaya et al., 2015)	2015	**P-Selectin (SELP)**: pathway associated with persistent antibody response. **miR-424:** regulator of the interferon response post vaccination	Identified pathways involved in a more long-term, durable response to influenza immunization. Identified interferon response and inflammatory markers that differ according to age. Persistent inflammatory state in elderly may explain immunosenescence.
Systems biology of immunity to MF59-adjuvanted vs. nonadjuvanted trivalent seasonal influenza vaccines in early childhood (Nakaya et al., 2016)	2016	**Module 75**: “antiviral interferon signature.” **Module 165**: “enriched in activated dendritic cells”	Identified modules that were correlated with strong HAI response post-vaccination with adjuvant.
Systems biology of vaccination for seasonal influenza in humans (Nakaya et al., 2011)	2011	**XBP-1:** corresponds with HAI response	Identified molecular signatures that correlated with B cell response to vaccinations, and showed how these can be used to predict vaccine response.
Global analyses of human immune variation reveal baseline predictors of post-vaccination responses (Tsang et al., 2014)	2014	PBMC subpopulation frequencies (baseline)	Described baseline characteristics that can be used to predict serologic response to influenza vaccines.
Apoptosis and other immune biomarkers predict influenza vaccine responsiveness (Furman et al., 2013)	2013	**APO module:** involved in apoptosis	Described a positive association between two gene modules involved with apoptosis and vaccine response to influenza.
Differences in antibody responses between trivalent inactivated influenza vaccine and live attenuated influenza vaccine correlate with the kinetics and magnitude of interferon signaling in children (Cao et al., 2014)	2014	**IFN-related modules:** M1.2, M3.4, M5.12, encode for IFN-inducible proteins. Overexpressed day 1 post-vaccination for the TIV group only, and more prominent in younger children.	Identified transcriptional patterns post-vaccination demonstrating that vaccines induced expression of interferon-related genes, which also was associated with antibody production.
Early patterns of gene expression correlate with the humoral immune response to influenza vaccination in humans (Bucasas et al., 2011)	2011	**494-gene signature** (mediates interferon response): positive correlation with antibody response to vaccination	Described a signature that corresponded to antibody response to the trivalent influenza vaccine.
Integrative genomic analysis of the human immune response to influenza vaccination (Franco et al., 2013)	2013	20 genes identified with correlation between transcriptional and antibody responses to vaccination, which influence the immune response to vaccine: **TAP2, SNX29, FGD2, NAPSA, NAPSB, GM2A C1orf85, JUP, FBLN5, CHST13, DIP2a, PAM, D4S234E, C3AR1, HERC2, LST1, LRRC37A4, OAS1, RPL14, DYNLT1**	Identified potential predictive biomarkers that can describe vaccine response. Many of the genes described are involved in membrane trafficking, antigen processing and antigen presentation.

Systems biology approaches have also been used to study human influenza infection, mostly in human cell culture, in order to better define pathogenesis, virulence factors, and immune responses. For example, transcriptomic responses to a human tissue culture cell line infected with various strains of influenza were examined by Josset et al. (2014). By analyzing transcriptomic responses to Influenza A Virus (IAV), the authors described that the avian H7N9 had further adapted to the human host. They also were able to test various therapeutics *in vitro*, and demonstrated antiviral responses associated with gene expression alterations. Other authors have studied proteomics in macrophages and monocytes of the lung after IAV infection. These studies showed interferon and TNF-alpha expression in response to infection, as well as pathways leading to secretions of specific proteins and antiviral cytokines (Lee et al., 2009; Lietzén et al., 2011; Cypryk et al., 2017). Ultimately, these findings demonstrate that macrophages could be used as a biomarker to determine the severity of influenza infection.

### Applying Systems Biology to the Human Influenza Challenge Model

To continue to advance our understanding of influenza, the techniques of systems biology should be furthered applied to the influenza challenge model. Very few studies have been published that use both techniques in concert. An influenza human challenge study was conducted by Woods in collaboration with Retroscreen Virology (London, UK), with pre-vaccination and post-vaccination RNA extracted from volunteers to examine gene expression, with the purpose of exploring new diagnostic options (Woods et al., 2013). Twenty-four healthy volunteers were inoculated with A/Brisbane/59/2007 (H1N1), and 17 were inoculated with A/Wisconsin/67/2005 (H3N2). The peripheral blood transcriptome was then analyzed at several time points over the course of 7 days. The results showed that peripheral blood gene expression after infection had a distinct signature that was specific to either H1N1 or H3N2 infection. Importantly, these genomic signatures were able to identify infected individuals before they manifested symptoms or had only mild, non-specific symptoms. This early recognition could lead to earlier administration of antivirals and have a more significant impact on lessening the severity of influenza illness. Sobel Leonard et al. used the same 17 participants inoculated with H3N2 from Woods' trial to evaluate evolution of the influenza virus at the time of transmission (Sobel Leonard et al., 2016). Using deep sequencing techniques, the nasal washings of the participants inoculated were compared with the viral stock and the reference strain (A/Wisconsin/67/2005) used to create the stock virus. They found that in acute infection in the human host, the influenza virus populations can undergo rapid viral evolution and changes, which largely occurs during a transmission bottleneck effect that is highly selective. Sobel Leonard et al. were able to further characterize these findings by describing viral evolution within the host and identifying genetic selection factors (Sobel Leonard et al., 2017).

A recent study by Jochems et al investigated the role of influenza infection leading to secondary bacterial pneumonia, using a “double” experimental human challenge model and systems biology approaches (Jochems et al., 2018). Subjects were first inoculated with human type 6B *Streptococcus pneumoniae* (Spn) to simulate carriage, which is an important pre-requisite of clinical pneumococcal pneumonia. The authors then gave the participants live attenuated influenza virus (LAIV) to simulate influenza infection. Using systems biology approaches of nasal secretions, they were able to identify specific immune mediators that control Spn carriage, and determine how influenza infection can affect these pathways. The results showed that LAIV increased the carriage density of Spn by impairing degranulation of nasal neutrophils and decreasing the recruitment of monocytes, which are two mechanisms essential for bacterial clearance. Although the study has limitations—only one pneumococcal serotype (6B) was used, and the influenza challenge was with LAIV, not wild-type influenza—the findings are important and highlight how the innate immune system is involved in Spn carriage and clearance, and how pre-existing viral infections can negatively affect immune-mediated control of infection. This study also underscores the power of applying systems biology approaches to the human challenge model. The granular details and elucidation of immune mechanisms were only able to be determined by integrating these two techniques.

## Conclusions

There is an urgent need to utilize novel platforms that can lead to the development of more effective vaccines and therapeutics for influenza, which continues to cause a significant burden of disease. Human challenge models have been successfully used for centuries. With advancing technologies and new methods to investigate the host-pathogen interaction, human challenge studies will be essential for progress, and can be performed in a safe and ethical manner. Furthermore, systems biology (e.g., transcriptomics, metabolomics, proteomics, lipidomics, etc.) allows for fundamental changes and patterns of the human immune system to be dissected. Harmonizing these two modalities is very promising; future studies should address using systems biology in a human challenge model to identify important gaps in our knowledge of influenza pathogenesis, and identify essential pathways involved in producing effective immune responses to vaccination. The ultimate goal would be to use these methods in concert to discover novel therapeutics, and potentially even lead to the development of a universal influenza vaccine.

## Author Contributions

AS and NR drafted the article. AS contributed to the collection and assembly of data, and the analysis and interpretation of the data. ND, AM, EA, and NR critically revised the article. ND contributed to the revision of the article and provided intellectual content for the ethics section.

### Conflict of Interest Statement

The authors declare that the research was conducted in the absence of any commercial or financial relationships that could be construed as a potential conflict of interest.
